# Oral‐cancer knowledge, practice, and attitude assessment of dentists in Upper Egypt: A cross‐sectional study

**DOI:** 10.1002/cre2.160

**Published:** 2019-03-06

**Authors:** Nagwa Mohmmad Ali Khattab, Ahmad Abdel Hamid Elheeny, Ghada Aslman Tony

**Affiliations:** ^1^ Paediatric and Community Dentistry Department, Faculty of Dentistry Minia University Minia Egypt

**Keywords:** attitude, dentists, Egypt, knowledge, oral cancer, practice

## Abstract

The aims of this study were to investigate the knowledge, attitude, and practice of dentists in Upper Egypt with regard to oral cancer (OC). Out of 1,200 licensed dental practitioners list, 424 dentists were randomly selected from three governorates in Upper Egypt (Minia, Assuit, and Sohag). The sample size was calculated using the equation considering the knowledge of dentists about OC, confidence level, and margins of error; then, an additional number of dentists were added to guard against nonresponse. Data were collected by face‐to‐face interview using 44 items divided into four sections; first part demonstrates sociodemographic. The second part concerned with the knowledge about OC clinical presentation and diagnosis (12 multiple‐choice questions) as well as its risk factors (17 close‐end questions). The third part consists of six questions focused on the practice of participants, and finally, the last part measures attitude of dentists. The chi‐square test was used to compare between the two or more proportions. A correlation was used for describing the relationship or association between two mutually numerical dependent variables. *p* < 0.05 was considered to indicate significance. Rate of response was 94.3%. The overall knowledge level in the current study was 31.8%. The awareness about OC risk factors was high especially, smoking tobacco and alcohol consumption. Also, over 80% of dentists identified family history and human papillomavirus (HPV) as risk factors. Only 37.5% of participants performed regular screening of oral mucosa, and 26.5% reported lymph‐node examination. Regarding attitude, only one quarter of dentists attended continuing educating programs about OC. A statistically significant relation (*p* < 0.0001) between knowledge level and most demographic variables was detected. There was a fair positive correlation (*r* = 0.47) between practice and knowledge scores. A predominant trouble among dentists in Upper Egypt was regarding OC knowledge and practice. Continues education and training programs are highly recommended.

## 1 INTRODUCTION

Oral cancer (OC) is one of the global diseases, which occupies the eighth rank among different types known of cancers (Petersen, Bourgeois, Ogawa, Estupinan‐Day, & Ndiaye, 2005; Razavi, Zolfaghari, Foroohandeh, Doost, & Tahani, 2013). Annually, the estimated number of OC new cases exceeds 300.000 cases (Al‐Jaber, Al‐Nasser, & El‐Metwally, 2016). Although the diverse distribution of the disease incidence worldwide, the number of new cases demonstrates increase in developing countries (Lopez‐Jornet, Camacho‐Alonso, & Molina‐Minano, 2010). This disease is responsible for global mortalities about 130.000 people every year (Petti & Scully, 2007). In Egypt, the incidence rate of OC is approximate ranges from 1.4 to 2 per 100.000 persons (Ferlay et al., 2015). Above 90% of all cancers of the oral cavity is oral squamous cell carcinoma (OSCC; Kujan et al., 2006; Llewellyn, Johnson, & Warnakulasuriya, 2001). Many risk factors for OC have been described, but smokeless and smoking tobacco, alcohol, and viral infection especially human papillomavirus (HPV) are among the fundamental factors for disease occurrence (Johnson et al., 2011; Somatunga et al., 2012). The diagnosis of OC in the early stage and subsequently early intervention with the required therapy have a positive impact on increasing the rate of survival up to 5 years (Lopez‐Jornet et al., 2010; Seoane et al., 2016; Tax et al., 2017). Fortunately, OC is one of the disease that can be discovered in the early stage through routine visual and tactile inspection of oral mucosa (Colella, Gaeta, Moscariello, & Angelillo, 2008). However, most of the cases are recognized in the late stages of the disease (III and IV; Esmaelbeigi et al., 2014; Francisco et al., 2016; Guneri & Epstein, 2014; Lopez‐Jornet et al., 2010). Therefore, OC prevention predominating relies on the dentists, and this can be achieved by raising the awareness of oral health‐care workers regarding OC signs and symptoms as well as its contributing factors (Canto, Horowitz, Drury, & Goodman, 2002; Monteiro, Salazar, Pacheco, & Warnakulasuriya, 2012). Another important aspect of prevention of OC is improving the dentist's level of practice and encouraging routine inspection of the oral mucosa of the whole patients especially, smokers or alcohol drinkers (Lopez‐Jornet et al., 2010). Furthermore, taking a biopsy or judicious referral to specialists for suspicious lesions is beneficial (Alonge & Narendran, 2003, 2004; Colella et al., 2008; Patton, Elter, Southerland, & Strauss, 2005). Despite several surveys that have been conducted to evaluate dental health‐care workers' knowledge, attitude, and practice about OC in different places all over the work, there is still a need for more similar studies (Colella et al., 2008). In Egypt, up to our knowledge, the number of studies regarding dentists' awareness and behavior towards OC is limited particularly large demogeographic area like Upper Egypt. Therefore, the current survey was designed to evaluate the knowledge, attitude, and practice of dentists about OC in Upper Egypt.

## 2 AIMS

The aims of this study were to investigate the knowledge, attitude, and practice of dentists in Upper Egypt with regard to OC.

## 3 SUBJECTS AND METHODS

After the permission from the Ethics Committee of Faculty of Dentistry, Minia University, is obtained, this observational cross‐sectional analytical survey was conducted during the period from August 2016 to January 2018.

## 4 STUDY SAMPLE

Before the study has started, the number of recorded licensed dentists in dentists association in each governorate was 1,200 dentists (450 dentists in Minia, 425 in Assuit, and 325 in Sohag). The study included 424 dentists who were randomly selected from the lists of licensed dental practitioners in three governorates in Upper Egypt (Minia, Assuit, and Sohag) using the computer‐generated simple randomization.

The sample size was calculated using the equation considering the knowledge of dentists about OC, confidence level, and margins of error; then, an additional number of dentists were added to guard against nonresponse. The used equation is *n* = *t* ^2^ × *p* (1 − *p*)/*m* ^2^ where *n* is the required sample size, *t* is the confidence interval at 95% (standard value of 1.96), *p* is the estimated dentists knowledge (50%), and *m* is the margin of error at 5% (standard value of 0.05).

## 5 DATA COLLECTION

Data were collected by using a 44‐item anonymous questionnaire to ensure confidentiality. Four hundred forty questionnaires (16 for the pilot study and 424 for the main study) were prepared in English and assessed and revised by a specialist, which have been used for conducting face‐to‐face interview. The questionnaire was pretested by conducting a pilot study with 16 dentists. After the pretest data were analyzed, Cronbach's *α* correlation coefficient, *α* = 0.91, indicating the survey had a high degree of internal consistency. The results of a pilot study were not included in the results of the current study. The time of the interview was about 20 min. The questionnaire consisted of four parts. First part demonstrates sociodemographic and work characteristics data of the participants that were covered through six items including age, gender, school and date of graduation, experience years, last academic degree, and specialty. The second part concerned with the knowledge about the OC clinical presentation and diagnosis (12 multiple‐choice questions) as well as its risk factors (17 close‐end questions). For each question, a score of either 0 = *incorrect* or 1 = *correct* was obtained. A total score of nine or higher correct responses out of the 12 questions (75%) were considered high OC level of knowledge (Khakbaz et al., 2017). The third part consists of six questions focused on the practice of the participants. The total score of practice was classified either good when at least four questions have positive answers. The last part measures the attitude of dentists and composed of six close‐ended questions. Attitude is considered favorable when a positive response was recorded for at least one half of the questions.

The questionnaire used in this study was long. Therefore, it is available at https://drive.google.com/open?id=1AmUSmCiCDm8z0At55aG8IZ8Fx9kOm2wZ.

## 6 STATISTICAL METHODS

Statistical Program Statistical Package for the Social Sciences (SPSS) 19 was used for data entry and analysis. Quantitative data were presented by mean and standard deviation, whereas qualitative data were presented by frequency distribution. The chi‐square test was used to compare between two or more proportions. A correlation was used for describing the relationship or association between two mutually numerical dependent variables. *p* < 0.05 was considered to indicate significance.

## 7 RESULTS

**Figure 1:**
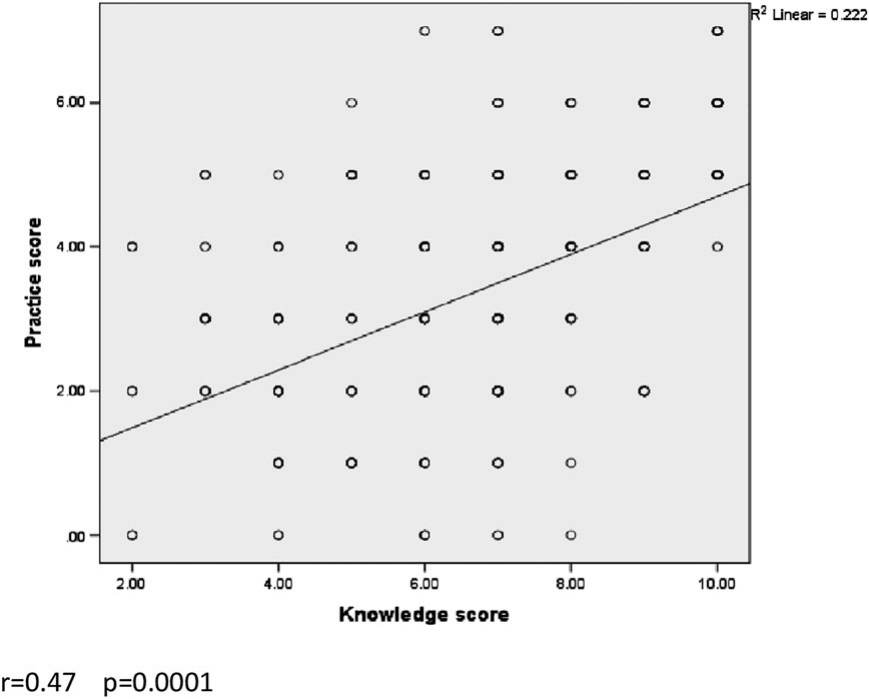
Correlation between scores of knowledge level and practice of participants

Out of 424 questionnaires, 400 fulfilled questionnaires were adopted for statistical analysis whereas 24 incomplete questionnaires have been excluded. The response rate in this study was high, 400 (94.3%). Approximately about 284 (71%) of dentists enrolled were working in general hospitals of Egyptian Ministry of Health at the time of study performing. Most of the participants are general dental practitioners, below the age of 35 years, and work for more than 6 hr daily. There is no sex predilection (Table 1). Although the overall knowledge level in the current study was 127 (31.8%), which considered low (Table 2), the awareness of our dentist about OC risk factors was high as all of them recorded that smoking, tobacco, and alcohol consumption and over 80% mentioned that family history, HPV, poorly fitted dentures, sun exposure, and consumption of hot beverages and foods are risk factors. The participants' practice evaluation clarified that only 106 (26.5%) of dentists' behaviors were good. Practice characteristics such as routine screening of oral mucosa and lymph‐node examination were only done by 150 (37.5%) and 106 (26.5%), respectively, (Table 3). More than 206 (50%) of the respondents demonstrated a favorable attitude, and 79% of them thought that OC should be a part of the routine examination. Only 156 (39%) of them convinced that they were qualified in performing diagnostic procedures. Also, 116 (29%) of the participants considered that the university provided training in OC examination during their undergraduate program, and 100 (25%) attended a continuing education program. However, 336 (84%) of dentists were interested in attending education courses on OC in the future. There was a statistically significant relationship between knowledge level and all demographic variables except the gender, *p* < 0.0001. Also, a statistically significant difference was obvious between dentists' practice and age, experience years, scientific degree, university of graduation, and working hours (Table 4). The correlation between practice and knowledge level was measured and showed fair positive correlation *r* = 0.47 between knowledge and practice scores (Figure 1).

**Table 1:** Distribution of demographic features of participants

Demographic features	Frequency (percent)
Health‐care facility	
General hospitals	240 (60)
Dental clinics	80 (20)
Scientific days	80 (20)
Age groups	
24–30	232 (58)
31–35	52 (13)
36–40	50 (12.5)
41–45	20 (5)
46–50	38 (9.5)
51–55	8 (2)
Gender	
Male	206 (51.5)
Female	194 (48.5)
Experience years	
1–5	148 (37)
6–10	118 (29.5)
11–15	44 (11)
16–20	66 (16.5)
21–25	12 (3)
26–30	12 (3)
Last scientific degree	
General Dental Practitioner (GDP)	332 (83)
Diploma/master degree	68 (17)
University of graduation	
Public	290 (72.5)
Private	110 (27.5)
Hours of work per day	
Less than 6	146 (36.5)
More than 6	254 (63.5)

**Table 2:** Distribution of knowledge level about common clinical features and diagnostic procedures

	Correct answers	Incorrect answers
Knowledge variables	Frequency	Frequency
	Percent	Percent
Clinical presentation and diagnostic issues		
Most common type of OC	329 (82.25)	71 (17.75)
Predominant age group	320 (80)	80 (20)
More common gender	220 (55)	180 (45)
Common condition associated to OC	238 (59.5)	162 (40.5)
Initial OC most common aspect	292 (73)	108 (27)
Most frequent anatomical region	312 (78)	88 (22)
Most frequent stage of diagnosis in Egypt	162 (40.5)	238 (59.5)
Relation of swelling size‐related OC stage	96 (24)	304 (76)
Characteristic cervical lymph‐node metastases	304 (76)	96 (24)
Familiar method of OC diagnosis	305 (76.25)	95 (23.75)
The best technique to confirm the diagnosis	122 (30.5)	278 (69.5)
Survival rates following early OC detection	256 (64)	144 (36)

*Note*. OC: oral cancer.

**Table 3:** Distribution of participants' practice regarding oral cancer (OC)

Practice variables	Frequency (percent)	
	Good practice	Poor practice
Routine examination of every patient oral‐mucosa routinely	150 (37.5)	250 (62.5)
Oral‐mucosa screening of high‐risk categories patients	157 (63)	92 (37)
Routine lymph‐node palpation	106 (26.5)	294 (73.5)
Tobacco and alcohol recording in personal history	194 (48.5)	206 (51.5)
Patient advise about OC risk factors	168 (42)	232 (58)
Take biopsy for suspicious lesions	110 (27.5)	290 (72.5)
Referral of a patient to which specialty	184 (46)	216 (54)

**Table 4:** The relation between knowledge, practice, and attitude and demographic variables

Demographic variables	Knowledge		Practice level		Attitude	
	Low	High	Poor	Good	Unfavorable	Favorable
	(*n* = 273)	(*n* = 127)	(*n* = 294)	(*n* = 106)	(*n* = 194)	(*n* = 206)
	N (%)	N (%)	N (%)	N (%)	N (%)	N (%)
Age groups						
24–30	192 (82.8)	40 (17.2)	186 (80.2)	46 (19.8)	108 (46.6)	124 (53.4)
31–35	34 (65.4)	18 (34.6)	34 (65.4)	18 (34.6)	20 (38.5)	32 (61.5)
36–40	31 (62)	19 (38)	30 (60)	20 (40)	38 (76)	12 (24)
41–45	7 (35)	13 (65)	14 (70)	6 (30)	8 (40)	12 (60)
46–50	8 (21.1)	30 (78.9)	24 (63.2)	14 (36.8)	16 (42.1)	22 (57.9)
51–55	1 (12.5)	7 (87.5)	6 (75)	2 (25)	4 (50)	4 (50)
	χ^2^ = 84.4^a^	*p* ˂ 0.0001	χ^2^ = 13.9	*p* = 0.02	χ^2^ = 18.8	*p* = 0.002
Gender						
Female	135 (65.5)	71 (34.5)	152 (73.8)	54 (26.2)	102 (49.5)	104 (50.5)
Male	138 (71.1)	56 (28.9)	142 (73.2)	52 (26.8)	92 (47.4)	102 (52.6)
	χ^2^ = 1.4	*p* = 0.2	χ^2^ = 0.02	*p* = 0.9	χ^2^ = 0.2	*p* = 0.7
Years of experience						
1–5	129 (87.2)	19 (12.8)	116 (78.4)	32 (21.6)	70 (47.3)	78 (52.7)
6–10	86 (72.9)	32 (27.1)	90 (76.3)	28 (23.7)	56 (47.5)	62 (52.5)
11–15	25 (56.8)	19 (43.2)	26 (59.1)	18 (40.9)	22 (50)	22 (50)
16–20	29 (43.9)	37 (56.1)	46 (69.7)	20 (30.3)	36 (54.5)	30 (45.5)
21–25	4 (33.3)	8 (66.7)	8 (66.7)	4 (33.3)	6 (50)	6 (50)
26–30	0 (0)	12 (100)	8 (66.7)	4 (33.3)	4 (33.3)	8 (66.7)
	χ^2^ = 78.8	*p* < 0.0001	χ^2^ = 8	*p* = 0.2	χ^2^ = 2.3	*p* = 0.8
Last scientific degree						
General Dental Practitioner (GDP)	264 (79.5)	68 (20.5)	227 (83.4)	55 (16.6)	164 (49.4)	168 (50.6)
Diploma/master degree	9 (13.2)	59 (86.8)	17 (25)	51 (75)	30 (44.1)	38 (55.9)
	χ^2^ = 111.4	*p* < 0.0001	χ^2^ = 95.5	*p* < 0.0001^b^	χ^2^ = 4	*p* = 0.5
University of graduation						
Public	70 (21.1)	220 (75.9)	229 (79)	61 (21)	135 (46.6)	155 (53.4)
Private	57 (51.8)	53 (48.2)	65 (59.1)	45 (40.9)	59 (53.6)	51 (46.4)
	χ^2^ = 26.9	*p* < 0.0001	χ^2^ = 15.2	*p* < 0.0001	χ^2^ = 1.3	*p* = 0.2
Work hours/day						
6	137 (93.8)	9 (6.2)	140 (95.9)	6 (4.1)	77 (52.7)	69 (47.3)
˃6	136 (53.5)	118 (46.5)	154 (60.6)	100 (39.4)	117 (46.1)	137 (53.9)
	χ^2^ = 67.6	*p* < 0.0001	χ^2^ = 57.4	*p* < 0.0001	χ^2^ = 1.4	*p =* 0.2

* *p* ˂ 0.0001; χ^2^ =chi‐squared test.

## 8 DISCUSSION

This study type is an observational cross sectional. An anonymous questionnaire was used in this quest, and this encouraged many participants to write down their actual behavior regarding the different questionnaire items. This had an impact on reducing the effect of information bias. Several methods were used in similar investigations such as email, postal service, or phone interviews, but the one‐to‐one interview has been chosen to perform this study because this method has been accredited to achieve a high quality of data and obtain more accurate results (Holman et al., 2018). Moreover, a personal interview may explain the high response rate of the current study, which was too close to studies performed in Iran and showed about 92% response rate (Mehdizadeh, Seyed Majidi, Sadeghi, & Hamzeh, 2014; Razavi et al., 2013). In contrast, several previously published articles in Brazil, Japan, Australia, and Spain showed a much lower response rate of less than 10% due to the dependence on sent electronic mails (Haresaku, Makino, Sugiyama, Naito, & Marino, 2018; Lopez‐Jornet et al., 2010; Pavao Spaulonci, Salgado de Souza, Gallego Arias Pecorari, & Lauria Dib, 2018). It was found that 82.25% of the dentists identified OSCC as the most common cancer of the oral cavity. This result was in agreement with published results of Motallebnejad and Hedayati, Mehdizadeh et al., and Eltelety et al. (Eltelety, Hassan, Kassimi, Qahatani, & Mohamed, 2014; Mehdizadeh et al., 2014; Motallebnejad & Hedayati, 2006). There was not a big difference between positive answers to the common age of OC, the initial aspect of OC, leukoplakia as a common condition associated with OC, and the characteristics of metastasized lymph nodes among our participants, and surveys were done in Italy, Brazil, and Iran (Colella et al., 2008; Pavao Spaulonci et al., 2018; Razavi et al., 2013). There was a wide variation among studies regarding the common site of the OC. In the current study, the tongue was selected by 78% of dentists, which is comparable with Kujan et al. who reported 85% correct answers (Kujan et al., 2014). On the other hand, an only 34% dentists in Jeddah in Saudi Arabia identified the correct answer (Eltelety et al., 2014). When asking about the OC common stage in Egypt, 76.25% of our participants identified the stage whereas only 24% of respondents awarded the relation between the size of the swelling and OC stage. These results were close to findings of Al‐Maweri et al. in Saudi Arabia, but 74.6% of dentists in a Brazilian in the study identified the relation of size and OC stage (Al‐Maweri et al., 2015; Pavao Spaulonci et al., 2018). The awareness of our dentist about OC risk factors was high as all of them recorded smoking tobacco and alcohol consumption and over 80% mentioned that family history, HPV, poorly fitted dentures, sun exposure, and consumption of hot beverages and foods are risk factors. These findings come inconsistency with a verity of surveys established in Spain, Italy, Brazil, Yemen, the United Kingdom, and the United States (Alaizari & Al‐Maweri, 2014; Boroumand, Garcia, Selwitz, & Goodman, 2008; Carter & Ogden, 2007; Colella et al., 2008; Horowitz, Drury, Goodman, & Yellowitz, 2000; Lopez‐Jornet et al., 2010; Patton et al., 2005; Pavao Spaulonci et al., 2018). Less than 40% of dentists considered lower consumption of fruits and vegetables, and only 23.8% had a positive response towards oral sex related to HPV transmission as OC risk factors. This spots the light on some OC risk factors that may be neglected during educational courses. Furthermore, knowledge about OC and its factors needs continuous updating. There was a positive correlation between all of these demographic variables and the level of knowledge of the participants. These findings were in contrast to a study performed in Brazil that pointed out the difference in knowledge between senior and junior dental clinicians for junior clinicians. Explanation of this situation could have more than one aspect, such as the shortage of undergraduate curricula of dental schools related to OC disease. Moreover, the attitude of participants played an important role in such situation like attending courses or conferences about OC and finally the design of the current study represented by its inclusion and exclusion criteria. Only 37.5% of our dentists reported that they perform a routine oral examination during dental visits. This is an indication of inadequate practice when compared with the study done in Spain by Seoane et al. and that is done in Cairo by Labib et al. (Labib, Elraghi, Shoman, & Othman, 2012; Seoane et al., 2016) as more than one half of the respondents in these studies reported that they were conducting the routine oral examination. Moreover, studies of Gajendra et al. and Vazquez‐Mayoral et al. recorded higher percentages than the current study; they were 85% and 52%, respectively, (Gajendra, Cruz, & Kumar, 2006; Vazquez‐Mayoral, Sanchez‐Perez, Olguin‐Barreto, & Acosta‐Gio, 2008). This could be considered as logical consequences, for weak knowledge level of our participants or their decision on the full‐mouth screening was influenced by the patient complaining of an oral health problem. There is a problem in response to most of the practice items, especially routine lymph‐node examination and qualified training to take a biopsy of the suspicious lesion (27%). This reflects the conviction of most dentists that lymph‐node examination performed only when the patient complains as well as a clear deficiency in training program availability. Only 25% of the dentists attended continuing educating programs, and 29% considered that the university provided satisfied training in OC examination during their undergraduate programs. These findings were compared with a study conducted in Iran by Mehdizadeh et al. (Mehdizadeh et al., 2014). In contrast, Pavao‐Spaulonci et al. reported that only 13% of dentists never attended any OC events before. This alarmists that the conferences for graduate dentists are not enough as well as university courses have a shortage regarding OC training (Pavao Spaulonci et al., 2018). In the current study, there was a statistically significant difference of knowledge level among dentists in relation to their age, years of experience, last scientific degree, and hours of work. There is a positive proportional relationship between all of these demographic variables and the level of knowledge of the participants. These findings were in contrast to a study performed in Brazil that pointed out the difference in knowledge between senior and junior dental clinicians for junior clinicians. Explanation of this situation could have more than one aspect, such as the weakness of undergraduate curricula of dental schools related to OC disease. Moreover, the attitude of participants played an important role in such situation like attending courses or conferences about OC and finally the design of the current study represented by its inclusion and exclusion criteria. In this study, there was a significant difference between knowledge level among age groups and years of experience (*p* < 0.0001). This comes in accordance with the study done in southern California by Forrest et al. as they found a significant difference among age groups and years of experience (Forrest, Drury, & Horowitz, 2001). Regarding the practice of dentists in this study, there was a statistically significant difference in their practice and their age, last scientific degree, and hours of work. This direct proportional relationship is a logical consequence of the participants' level of knowledge. In another aspect, the correlation between participants' attitude and their demographic data was statistically insignificant except for different age groups.

## 9 CONCLUSIONS

It can be concluded from the results of this study that dentists in Upper Egypt have obvious problems in the knowledge of OC diagnosis. Routine screening of oral mucosa is another poor sign of practicing among dentists. Unfortunately, these shortages in knowledge and practice are a real threat against prevention and early detection of the disease and subsequently reducing its burden. Practicing was significantly improved in correlation with age, university of graduation, and hours of daily work. The good news was that most of the respondents were interested in improving their knowledge and practice levels. It is highly recommended to apply continues education and training programs in the form of lectures, courses, or workshops through cooperation between universities especially, in Upper Egypt and Egyptian Ministry of Health. Revising and updating the current OC educational curricula in dental schools especially those in Upper Egypt.

## CONFLICT OF INTEREST

None declared.
